# Prevalence of gram negative bacteria causing community acquired pneumonia among adults in Mwanza City, Tanzania

**DOI:** 10.1186/s41479-020-00069-0

**Published:** 2020-08-05

**Authors:** Peter Kishimbo, Nyambura Moremi Sogone, Fredrick Kalokola, Stephen E. Mshana

**Affiliations:** 1 Department of Internal Medicine, Weill Bugando School of Medicine, Mwanza, Tanzania; 2 National Health Laboratory Quality Assurance and Training Centre, Dar es Salaam, Tanzania; 3 Department of Microbiology/Immunology, Weill Bugando School of Medicine, Mwanza, Tanzania

## Abstract

**Background:**

Community acquired pneumonia (CAP) in adults is still a common and serious illness in the sub-Saharan Africa. Identification of the pathogens is crucial in the management of CAP. This study was done to determine the common bacterial pathogens, treatment outcomes and associated factors for microbiological confirmed CAP among adults attending the Bugando Medical Centre and Sekou Toure hospital in the city of Mwanza, Tanzania.

**Methods:**

This was a hospital based cross sectional study involving patients with community acquired pneumonia attending Bugando Medical Centre and Sekou Toure regional Hospital. Demographic and other data were collected using standardized data collection tool. Sputum culture was done followed by identification of the isolates and antibiotics susceptibility testing.

**Results:**

A total of 353 patients were enrolled in the study. Out of 353 sputum samples, 265(75%) were of good quality. Of 353 non-repetitive sputum cultures, 72/353 (20.4, 95% CI: 16.2–24.6) were positive for the bacterial pathogens with five patients having more than one pathogen. Good quality sputa had significantly higher yield of pathogenic bacteria than poor quality sputa (26.1% vs.3.4%, *P* = 0.001). The majority 64 (83.1%) of the isolates were gram negative bacteria. Common bacteria isolated were *Klebsiella pneumoniae* 23/77(29.9%), *Streptococcus pyogenes* 10/77 (13.0%), *Pseudomonas aeruginosa* 9/77 (11.7%) and *Escherichia coli* 7/77 (9.1%). Of 23 *K. pneumoniae* isolates, 20/23 (87.0%) were resistant to ceftriaxone. Resistance to ceftriaxone was found to be associated with prolongation of CAP symptoms (*p* = 0.009).

**Conclusion:**

Gram negative bacteria resistant to ampicillin, amoxicillin/clavulanic acid and ceftriaxone were most frequently isolated bacteria among adults’ patients with CAP attending BMC and Sekou Toure hospital. Routine sputum culture should be performed to guide appropriate treatment of CAP among adults in developing countries.

## Background

Community-acquired pneumonia (CAP) according to British Thoracic Society is defined as an acute symptomatic infection of the pulmonary parenchyma which develops outside the hospital or nursing home as evidenced by a new infiltrate demonstrated on chest radiograph or auscultatory findings consistent with pneumonia [1]. CAP is increasing in the sub-Saharan Africa and is among the major cause of morbidity and mortality among adults [2–4]. CAP ranks third as the most common cause of death worldwide [5, 6]. CAP is prevalent in both developing and developed countries; however it is about five times more common in developing countries [6, 7].

Etiological causes of CAP vary significantly between countries [7], also there are variations within the same countries [8]. Etiological variations are due to irrational use of antibiotics, environmental pollution, advancement in diagnostic techniques/tools and increased awareness of the disease [9]. Diagnosis of CAP depends on different diagnostics methods; including chest radiography which is required to demonstrate new infiltrates, sputum and blood culture which can confirm the microbiological pathogens [8]. Microbiological studies of sputum (microscopic examination of sputum specimens and sputum culture) can yield the correct diagnosis of CAP in the majority of cases [8]. Different guidelines including those from Infectious Disease Society of America (IDSA) recommend 2 pre-treatment blood cultures as well as gram staining and culture of the sputum in the diagnosis of CAP [8].

Studies in Europe, United Sates and Asia have documented *Streptococcus pneumoniae* as the most isolated organism responsible for the CAP [10]. In the developing countries, despite CAP being the commonest reason for hospital visits, there are limited information regarding the etiological causes of CAP in these countries [11].

No prior study has been done at the Bugando Medical Centre (BMC) and Sekou Toure hospital to document the pathogens causing CAP. The current empirical treatment depends on the data from developed countries which might not be applicable in our setting. In the view of this burden and the increasing concern about irrational use of antibiotics, this study aimed at determining the etiological pathogens and susceptibility patterns of isolates causing community acquired pneumonia among adults attending Bugando and Sekou Toure hospital in Mwanza City, Tanzania.

## Methods

### Study design and site

This was a hospital based cross sectional study conducted in medical wards and medical outpatient departments of the Bugando Medical Centre (BMC) and Sekou Toure regional hospital between August 2015 and December 2015. The study included in-patients and outpatients’ adults aged 18 years and above who met inclusion criteria.

### Sample size calculation

The Kish Leslie [12] formula was used to calculate the required sample size using proportion from a study in Kenya which observed 65% of patients with CAP had defined etiology [13] giving a minimum sample size of 350 adult patients.

### Inclusion criteria and exclusion criteria

**Fig. 1:**
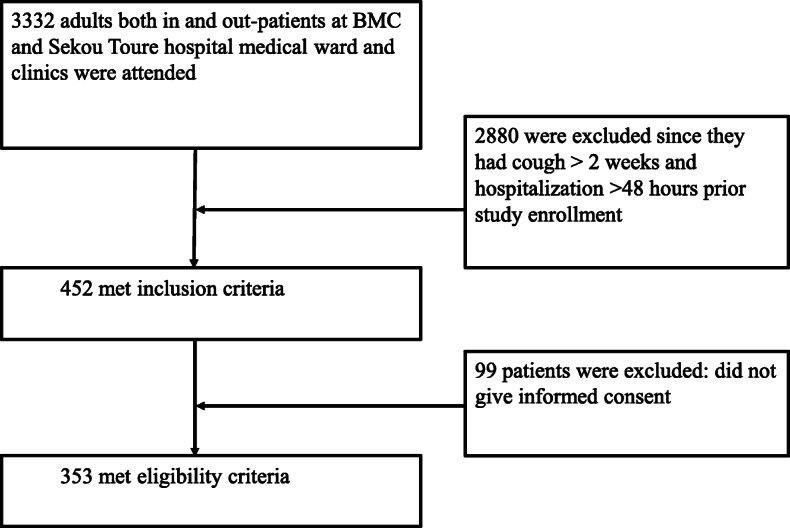
Flow chart regarding patient recruitment

Out- and in-patients adult aged 18 years or above attending at BMC and Sekou Toure regional hospital with productive cough and at least 2 symptoms of acute lower respiratory infections were included. These symptoms included: change in color of respiratory secretions in a patient with chronic cough, axillary temperature of > 37.5 °C or hypothermia 36.1 °C, chest discomfort or the onset of dyspnea, new chest clinical features (rales, wheezing, absence of breath sounds, egophony etc.) consistent with pneumonia demonstrated on auscultatory findings. The study excluded patients hospitalized for more than 48 h prior to enrollment in the study, patients who were discharged from the hospital within 2 weeks prior to study enrollment and those with cough of more than 2 weeks (Fig. 1).

### Data collection

Data were collected using standardized data collection tool. Physical examination included a single blood pressure measurement which was obtained with automated blood pressure, deluxe automatic blood pressure monitor (HoMedics Inc., China). Other examinations were respiratory rate and pulse rate, measured as previously described [14]. Oxygen saturation was recorded in percentage by pulse oximeter (Beijing Choice Electronic Tech. Co. Ltd) placed on finger for at least 5 min. All patients with no HIV results, were counseled for HIV testing following Tanzania HIV testing National algorithm [15].

All enrolled patients were provided with containers for sputum collection. Education was provided on how to collect quality sputum as described in Koneman’s Color Atlas and Textbook of Diagnostic Microbiology [16].

All patients were followed for 14 days through telephone to determine the cure rate (disappearance of symptoms). Patients were treated by the discretion of doctor, but antibiotics were switched basing on culture and susceptibility results.

### Sputum processing

Sputum specimens were processed according to BMC microbiology standard operation procedures. A BMC laboratory is accredited by the Southern African Development Community Accreditation Service (SADCAS) with a unique number MED 002. Specimens were examined both macroscopically and microscopically. Macroscopic appearance of the specimens were recorded i.e. saliva, purulent or bloodstained. Purulent portion of specimen was selected for microscopy studies and culture [17, 18]. The quality of sputa was assessed using Bartlett et al., criteria. The sputum with Q-score of more than 1, i.e. WBC more than 10 was considered acceptable and termed as good quality while a score of “0” or “– “was considered non-acceptable and termed as poor quality [19].

### Culture and identification

The purulent part of the sputum was inoculated on Chocolate agar plate, Blood agar plate and MacConkey Agar (OXOID, Hampshire, United Kingdom) and incubated in 5–10% CO_2_ for 24 h. Cultures were read after 24 h and in some cases after 48 h. Interpretations were recorded as semi-quantitative growth of all colonies (i.e. +/− to ++++). The significant growth of possible pathogens other than normal oral flora was considered as positive culture [20]. Pathogens were identified using in house biochemical and physiological properties as described previously [17, 21]. All pathogens were subjected to the susceptibility testing using disc diffusion methods as per Clinical and Laboratory Standard Institute [22]. *S. aureus* isolates with cefoxitin (30 μg) zone of inhibition of ≤21 mm were phenotypically considered as methicillin resistant *Staphylococcus aureus* (MRSA).

*Streptococcus pneumoniae* (ATCC 49619), *Haemophilus influenzae* (ATCC 49247/49766) and *Staphylococcus aureus* (ATCC 25923) were used as quality control strains.

### Data analysis

Data were entered into computer using Microsoft Excel. Data cleaning was done by EpiData version 3.1 (CDC, Atlanta, USA) according to codes given and finally analyzed using STATA version 13 (College Station, Texas, USA). Binary and categorical variables (age, sex, blood pressure, oxygen saturation and respiratory rate monitoring) were described as proportions and percentages and compared using Chi-Square and Fisher’s exact tests. Continuous variables were described as medians with interquartile ranges (IQR) and compared using Wilcoxon Rank Sum test. The primary outcome of this study was the culture positive for pathogens from expectorated sputum. The strength of association of the factors associated was determined using univariate logistic regression analysis to obtain odd ratio and its 95%CI. All variables with p-value of less than 0.05 at 95% confidence interval were considered to have statistical significant difference.

## Results

### Demographic and other characteristics of the study population

A total of 353 adults were included in the study (Fig. 1). The median age was 40(IQR; 32–52) years. A total of 181 (51.3%) participants were males. The majority of patients 322 (91.2%) were enrolled in the medical outpatient clinics. Ninety three (26.3%) of the patients had ever smoked tobacco containing products in their life. Diabetes Mellitus, heart diseases, chronic kidney disease and chronic obstructive pulmonary diseases (COPD) were reported in 40 (11.3%) of the enrolled patients. One hundred-twenty two (34.6%) of patients had history of antibiotics use in the past 7 days prior to the enrolment. None of the patients had ever been vaccinated for respiratory infections (Table 1).

**Table 1:** Baseline characteristics of 353 adult patients with pneumonia between August and December 2015 in Mwanza, Tanzania

Characteristics	Number (%) or Median (IQR)
**Sex**	
Male	181 (51.3)
Female	172 (48.7)
**Age (years)**	40 (32–52)
**Hospitalization**	
Inpatients (Less than 48 h.)	31 (8.8)
Outpatients	322 (91.2)
**Level of formal education**	
None	51 (14.5)
Primary school	228 (64.6)
Secondary school	64 (18.1)
University/College	10 (2.8)
**Ever Smoked tobacco products**	
No	260 (73.7)
Yes	93 (26.3)
**Taking of alcohol-containing beverage**	
No	189 (53.5)
Yes	164 (46.5)
**HIV status**	
Negative	217 (61.5)
Positive	136 (38.5)
**Co-morbidities**	
No	313 (88.7)
Yes	40 (11.3)
**Antibiotics use in the past 7 days**	
No	231 (65.4)
Yes	122 (34.6)
**Treatment**	
First line	265 (75.1%)
Second line	88 (24.9%)

In this study: First line antibiotics included Amoxicillin/clavulanic acid or Ampicillin and trimethoprim/ sulphamethoxazole, Second line antibiotics was either Ceftriaxone or Ceftazidime

### Bacterial pathogens and their susceptibility patterns

**Fig. 2:**
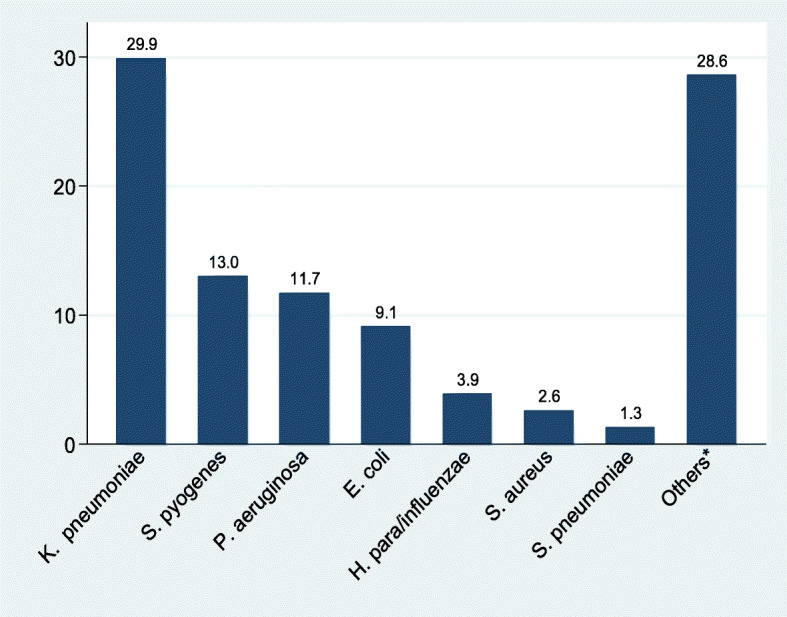
Bacterial isolates from 72 patients with positive sputum culture

Out of 353 non-repetitive sputum cultures, 72/353 (20.4, 95% CI) were positive for bacterial pathogens with 5(1.4%) patients (Table 2) having more than one isolate. The majority of the isolates were gram negative bacteria 64 (83.1%), Fig. 2. Out of 353 non-repetitive sputum samples, 265 (75.1%) were of good quality. Common bacteria isolated were *Klebsiella pneumoniae* 23/77(29.9%), *Streptococcus pyogenes* 10/77 (13.0%), *Pseudomonas aeruginosa* 9/77 (11.7%), *Escherichia coli* 7/77 (9.1%), *Haemophilus influenzae/parainfluenzae* 3/77 (3.9%), *Staphylococcus aureus* 2/77 (2.6%) and *Streptococcus pneumoniae* 1/77 (1.3%)*.* Other bacteria isolated were: *Citrobacter freundii* (3/22), *Enterobacter aerogenes* (3/22), *Klebsiella oxytoca* (1/22), *Moraxella* spp*.*(1/22), *Proteus mirabilis* (2/22), *Serratia marcescens* (1/22) and unidentified Gram Negative Rods (GNR) (11/22), Fig. 2.

**Table 2:** Patients with more than one isolate

Patient ID	Age (years)	Sex	Isolates	Outcome (symptoms disappeared after 14 days)
29	35	Female	*Klebsiella pneumoniae Escherichia coli*	Yes
46	30	Male	*Escherichia coli* Unidentified GNR	No
136	39	Male	*Pseudomonas aeruginosa* Unidentified GNR	No
271	31	Male	*Klebsiella pneumoniae Enterobacter aerogenes*	NO
278	76	Male	*Klebsiella pneumoniae Citrobacter freundii*	Yes

Of 23 *Klebsiella pneumoniae* isolates, 21(91.3%) were resistant to amoxicillin-clavulanic acid and 20 (87.0%) were resistant to ceftriaxone. *Escherichia coli* isolates were 100% resistant to ampicillin with 6/7 (85.7%) being resistant to ceftriaxone (Table 3). All *Staphylococcus aureus* isolates were susceptible to trimethoprim-sulfamethoxazole and none was phenotypically confirmed to be MRSA.

**Table 3:** Resistance rates for bacterial pathogens from adult patients with community acquired pneumonia between August and December 2015 in Mwanza, Tanzania

ISOLATE	AMP	CIP	CN	SXT	AMC	CRO	MEM	TET	P	E	VA	DA
*K. pneumoniae* (23)	100%)	17.4%	26.1%	43.5%	91.3%	87.0%	4.3%	NT	NA	NA	NA	NA
*E. coli* (7)	100%	28.6%)	14.3%	71.4%	85.7%	85.7%	0.0%	NT	NA	NA	NA	NA
*P. aureginosa* (9)	100%	50%	39%	100%	100%	90.0%	0.0%	NA	NA	NA	NA	NA
^*a*^*Others GNB* (25)	68.2%	9.1%	0.0%	40.9%	36.4%	77.0%	0.0%	NT	NA	NA	NA	NA
*Str. pyogenes* (10)	NA	10.0%	20.0%	100.0%	NA	NA	NA	10.0%	0.0%	10.0%	0.0%	0
*Str. pneumoniae* (1)	NT	NT	NT	NT	NA	NT	NT	0.0%	0.0%	100%	NT	NT
*S. aureus* (2)	NA	0	0	0	NA	NA	NA	100%	NT	50%	0.0%	0.0%

*NT* Not done, *NA* not applicable, *AMP* Ampicillin, *CIP* Ciprofloxacin, *CN* Gentamicin, *SXT* Trimethoprim/Sulphamethoxazole, *AMC* Amoxicillin/clavulanate, *CRO* Ceftriaxone, *MEM* Meropenem, *TET* Tetracycline, *E* Erythromycin, *VA* Vancomycin and *DA* Clindamycin

^a^*Citrobacter freundii, Enterobacter aerogenes, Klebsiella oxytoca, Moraxella spp., Proteus mirabilis, Serratia marcescens and un-identified Gram Negative Rods (GNR), Haemophilus influenzae/parainfluenzae*

### Factors associated with sputum culture positive

Out of 181 males, 41 (22.7%) were found to be sputum culture positive compared to 31/172 (18.0%) females (OR 0.8, 95%-CI, (0.4–1.3, *p* = 0.282). Those with history of antibiotics use in the past 7 days prior to enrollment, had low yield of sputum culture results compared to patients without prior antibiotics use(18.2% versus 24.6%, OR 1.5, 95% CI:0.9–2.5, *p* = 0.157). Sputum samples of good quality had ten folds increased chances of yielding positive sputum cultures as compared to poor quality sputum (OR 10.1, 95%CI;3.1–33.1, *P* = < 0.001) (Table 4).

**Table 4:** Factors associated with Sputum Culture Positive of 353 adult patients with pneumonia between August and December 2015 in Mwanza, Tanzania

Predictor	Sputum Culture		Univariate	
	Positive n (%)	Negative n (%)	OR[95%-CI]	***P***-value
**Sex**				
Male	41 (22.7)	140 (77.3)	1.0	
Female	31 (18.0)	141 (82.0)	0.8 [0.4–1.3]	0.282
**Age (years)**	39 (32.5–53)	40 (32–52)	1.0 [0.99–1.01]	0.950
**Ever smoked tobacco products**				
No	52 (20.0)	208 (80.0)	1.0	
Yes	20 (21.5)	73 (78.5)	1.1 [0.6–1.9]	0.757
**Alcohol-containing beverage**				
No	43 (22.8)	146 (77.2)	1.0	
Yes	29 (17.7)	135 (82.3)	0.7 [0.4–1.2]	0.240
**Co morbidities**				
No	62 (19.8)	251 (80.2)	1.0	
Yes	10 (25.0)	30 (75.0)	1.3 [0.6–2.9]	0.444
**Hospitalization in past 2 weeks**				
No	70 (20.1)	278 (79.9)	1.0	
Yes	2 (40.0)	3 (60.0)	2.6 [0.4–16.1]	0.291
**Antibiotics use in the past 7 days**				
No	42 (18.2)	189 (81.8)	1.0	
Yes	30 (24.6)	92 (75.4)	1.5 [0.9–2.5]	0.157
**HIV infection status**				
Negative	46 (21.2)	171 (78.8)		
Positive	26 (19.1)	110 (80.9)	0.87 [0.51–1.5]	0.637
**Quality of sputum sample**				
Poor	3 (3.4)	86 (96.6)	1.0	
Good	69 (26.1)	195 (73.9)	10.1 [3.1–33.1]	**< 0.001**

### Factors associated with uncommon pathogens

A total of 39(54.2%) of patients were infected by common pathogens (*K. pneumoniae*, *S. pyogenes*, *H. influenza*, *S aureus*, *S. pneumoniae, Moraxella* spp.*)* while 33(45.8%) were infected with uncommon pathogens *(Pseudomonas* spp*., E. coli, Citrobacter* spp.*, Enterobacter* spp*., Proteus* spp*., Serratia* spp*.,* and *un-identified* GNB). Out of 33 patients with uncommon pathogens, 12(36.4%) were HIV positive compared to 14/39(35.9%) who were HIV positive among those with common pathogens (*P* = 0.967). The sub-analysis between pathogens and antibiotic use in the past 7 days, revealed that, those with history of antibiotics use had higher positive culture due to uncommon pathogens than those who had no history of antibiotic use (18/30(60%) vs. 15/42(35.7%), *p* = 0.041). Regarding co-morbidities, no significant different was observed in distribution of pathogens among those with co-morbidities and those with no co-morbidities (28/62, 45.2% vs. 5/10, 50%).

### Factors associated with symptoms disappearance after 14 days follow up

Out of 72 patients with sputum culture positive, 45 (62.5%) presented with no symptoms after 14 days compared to 218(77.6%) of those who were culture negative (OR = 2 95%CI; 1.25–3.30, *p* = 0.010). Resistance to the second line antibiotic was associated with persistence of symptoms in the majority of cases. Further analysis revealed that, 14(82.3%) of those infected with gram negative isolates susceptible to ceftriaxone had their symptoms disappeared compared to only 17(44.7%) of those infected by ceftriaxone non susceptible isolates (*p* = 0.009) (Table 5).

**Table 5:** Factors associated with symptoms disappearance after 14 days follow up of 353 adults’ patients with pneumonia between August and December 2015 in Mwanza, Tanzania

Predictor	Symptoms disappeared		Univariate	
	YES, n (%)	NO n (%)	OR[95%CI]	***P***-value
**Sex**				
Male	134 (74.0)	47 (26.0)	1.0	
Female	129 (75.0)	43 (25.0)	1.1 [0.7–1.7]	0.835
**Age (years)**	40 (31–53)	42 (35–51)	1.0 [0.9–1.0]	0.527
**Sputum culture positive**				
Yes	45 (62.5)	27 (37.5)	1.0	
No	218 (77.6)	63 (22.4)	2 [1.25–3.30]	**0.010**
**Gram staining**				
Gram negative	37 (62.7)	22 (37.3)	1.0	
Gram positive	8 (61.5)	5 (38.5)	2 [0.66–10]	0.189
No organism	218 (77.6)	63 (22.4)	2 [1.1–3. 3]	**0.018**
**Treatment given**				
First line	232 (87.5)	33 (12.4)	1	
Second line	31 (64.6)	57 (35.4)	0.25 (0.12–0.55)	**< 0.001**
**Second line treatment antibiotics resistance**				
No	14 (82.3)	3 (17.7)	1.0	
Yes	17 (44.7)	21 (55.82)	0.17 [0.03–0.79]	**0.009**
**Co morbidities**				
No	234 (74.8)	79 (25.2)	1.0	
Yes	29 (72.5)	11 (27.5)	0.9 [0.4–1.9]	**0.758**
**HIV infection status**				
Negative	79 (74.5)	27 (25.5)		
Positive	101 (74.3)	35 (25.7)	1.0 [0.6–1.8]	0.963

## Discussion

In this hospital based cross sectional study, bacterial pathogens were isolated in 20.4% of the patients which is lower than the findings in others studies in Bangladesh 27.6% [23], South Ethiopia 40.0% [24], Iran 44% [25], Nicaragua 45.0% [26] and Nigeria 47.2% [27]. The difference could be explained by the fact that in those studies, the majority of patients enrolled were older than patients in present study. The bacterial CAP is more likely in older age than in young age group [28]. The absence of bacterial isolates in the majority of the patients in this study could be attributed by antibiotics use before presentation to hospital [29] as evidenced by the fact that, sputa from patients with history of antibiotic use had low yield. However; other studies have observed that despite comprehensive diagnostic work-up, causes of CAP in adults may remain undetermined in 40–60% of individuals even in the best centers [1, 30]. Other explanation could be that the etiology of CAP in this cohort could be viruses as observed in previous study that viruses might be confirmed as cause of CAP in 29% of adult patients [5].

As in previous studies high quality sputum had significantly more yield of pathogenic bacteria than poor quality sputum [31, 32]. The findings emphasize the importance of proper instructions to the patients to ensure high quality sputum specimens are obtained for microbiological investigations.

Gram negative bacteria were commonly isolated in the present study. Previous studies in Africa and elsewhere have reported similar results indicating a shift of pathogens trends [24, 33]. In contrast to studies in USA and Europe, only one patient in the present study was infected with *S. pneumoniae* [9, 23, 34, 35]. Self-medication of antibiotics in the study setting could explain this because the majority of *S. pneumoniae* strains are susceptible to erythromycin and amoxicillin which are commonly self-administered antibiotics [36, 37]. In addition, geographical variations of bacterial CAP in adults can contribute to the observed differences [7].

As far as antibiotics susceptibility patterns is concerned, the majority of the isolates in this study were highly sensitive to meropenem and ciprofloxacin. These findings are similar to other studies carried out in Estonia and Nigeria [38, 39]. Nevertheless, a significant proportion of isolates were resistant to ceftriaxone, amoxicillin-clavulanic and ampicillin. These antibiotics are widely used empirically to treat adults with CAP in our setting. The observed resistance rates to these antibiotics were higher than other studies elsewhere [23, 38]. The higher resistance could be due to irrational use of these antibiotics in our setting, as it was further observed that in these hospitals ceftriaxone, amoxicillin-clavulanic acid and ampicillin were commonly used to any patient with cough and fever regardless of the duration symptoms. The observation is further supported by the fact that those patients with history on antibiotic use were significantly more likely to be infected with uncommon pathogens such as *Pseudomonas* spp., *E coli*, *Citrobacter* spp., *Enterobacter* spp., *Proteus* spp. and *Serratia* spp. These pathogens tend to be more resistant than common pathogens. There is high antibiotics self-medication in Tanzania that might contribute to high rate of multidrug resistant gram-negative bacteria causing community acquired pneumonia [40–43]. Furthermore it should be noted that these patients were enrolled at regional and tertiary hospitals therefore have either visited lower facilities for treatment or community pharmacy for self-medication.

The high resistance rates to commonly used antibiotics is also supported by previous community studies in the city of Mwanza by Bahati et al [44] and Mshana et al [45] which identified a significant proportion of *Escherichia coli*, *Klebsiella pneumoniae* and other Enterobacteriaceae species (*Enterobacter* spp., *Proteus mirabilis* and *Proteus vulgaris*) being resistant to ceftriaxone, ampicillin, trimethoprim/sulfamethoxazole, amoxicillin/clavulanic and gentamicin. Recent review has shown that CAP due to multi-drug resistant is common and is associated with significant morbidity and mortality and it was emphasized that identification of these pathogens and patients at risk is very important in order to institute appropriate antibiotic treatment [46].

Positive sputum culture, gram stain with predominant gram negative bacteria and resistance to second line antibiotics were significantly associated with low rates of symptoms disappearance. Regarding gram staining and treatment outcome, findings of this study are similar to those reported by Liao X et al in Tianjian [47]. A study in Nigeria also observed that the resistance to ceftriaxone was associated with longer duration of hospital stay for hospitalized patients with CAP [38]. It should be noted patients who received second line treatment were more likely to have resistant pathogens because of previous antibiotic exposure.

Limitation of this study included: Failure to identify atypical bacteria because serological or molecular techniques were not available at the study setting. In addition, chest –x-ray and blood culture were not performed. However clear criteria were used to enrol the patients with suspected community acquired pneumonia and sputum culture [48] has been found to be useful in the diagnosis of community acquired pneumonia with limited usefulness of blood culture in relation to yield and cost [49].

## Conclusion

Gram negative bacteria are the commonest cause of CAP among adults in the city of Mwanza, north-western Tanzania. These isolates were significantly resistant to first and second line antibiotics commonly used in our setting. Resistance to commonly used antibiotics (cephalosporins) for empirical treatment of CAP in the region was associated with worse outcome in terms of symptoms disappearance. Based on these findings there is a need for an evidence based treatment guidelines for CAP in developing countries. In the absence of other advanced diagnostic techniques in developing countries and limited usefulness of blood culture, there is a need to emphasize sputa culture to guide antibiotic treatment for bacterial CAP in developing countries.

## Data Availability

The datasets used and/or analyzed during the current study available from the corresponding author on reasonable request.

## Abbreviations

ATCC: American type culture collection
BMC: Bugando Medical Centre
CAP: Community acquired pneumonia
CUHAS: Catholic University of Health and Allied Sciences
CLSI: Clinical Laboratory Standard Institute
CDC: Center for Disease control
COPD: Chronic Pulmonary Diseases
ESBL: Extended Spectrum beta-lactamases
MOPD: Medical outpatient Department
EMD: Emergence medicine department
